# Quantitative proteomics reveal the anti-tumour mechanism of the carbohydrate recognition domain of Galectin-3 in Hepatocellular carcinoma

**DOI:** 10.1038/s41598-017-05419-5

**Published:** 2017-07-12

**Authors:** Mingchao Wang, Fang Tian, Wantao Ying, Xiaohong Qian

**Affiliations:** Beijing Institute of Radiation Medicine, National Center for Protein Sciences Beijing, State Key Laboratory of Proteomics, Beijing Proteome Research Center, Beijing, China

## Abstract

Hepatocellular carcinoma (HCC) is a serious threat to human health. The carbohydrate recognition domain of Galectin-3 (Gal3C) has been reported to be an anti-tumour molecule. In this study, we aim to explore effects of Gal3C in HCC and its possible molecular mechanism with quantitative proteomics approach. We found that rGal3C stimulation could inhibit cell viability, migration and invasion of HepG2. After rGal3C stimulating, 190 proteins were differentially expressed. Eighty up-regulated proteins located mainly in extracellular exosome and involved in cell adhesion and metabolism, and 110 down-regulated proteins located in mitochondria and extracellular exosome, and related to processes of metabolism and oxidation-reduction. Of the differentially expressed proteins, CLU, NDRG1, CD166, S100A11 and Galectin-1 were carcinoma-related proteins affected by rGal3C. Potential receptors of rGal3C were explored by an UV cross-linking capture strategy. We showed that rGal3C could induce dephosphorylating of FAK/SRC. Blocking of the FAK/SRC pathway resulted in down-regulation of NDRG1. Immunofluorescence suggested that rGal3C could disrupt integrin clustering. Our study provides valuable insight into the anti-tumour mechanism of rGal3C in HCC on a proteomics level and is the first to reveal the possible mechanism involving integrin/FAK/SRC pathway and NDRG1. These results provide useful guidance of developing new therapies for HCC.

## Introduction

Hepatocellular carcinoma (HCC) is the most common malignant liver cancer in the modern world^1^. The occurrence of HCC involves complex induction factors, such as Hepatitis B virus (HBV), Hepatitis C virus (HCV), alcohol and tobacco^2^. In general, most HCC cases develop from liver cirrhosis (LC)^3^.

Tumourigenesis of HCC is regulated by a series of pathways, such as Wnt/β-catenin, Notch, EGFR, RAS/MAPK/ERK, AKT/mTOR and FAK/SRC^4–8^. HCC progress is also accompanied by changes in protein expression; thus, some proteins can be used as potential biomarkers for diagnosis and treatment^9^. Currently, alpha-fetoprotein (AFP) is still the most common serum marker for HCC diagnosis, and its fucosylated fraction, AFP-L3, is an even more sensitive tumour marker^10, 11^. Other biomarkers used for HCC detection include GPC3, Osteopontin, and Golgi Protein 73 (GP73)^12^. Recently, N-myc downstream-regulated gene 1 protein (NDRG1)^13^ and Activated leukocyte cell adhesion molecule (ALCAM, CD166)^14^ have been identified as novel tumour markers for clinical diagnosis of HCC.

Galectin-3 is a mammalian lectin that is up-regulated in HCC and involves in tumour progression and poor prognosis in HCC. Therefore, it may serve as a prognostic marker for HCC^15–19^. Intracellular Galectin-3 is mostly a monomeric soluble protein regulating apoptosis^20–24^ and phosphorylation of AKT in cytoplasm^25, 26^, and Wnt/β-catenin pathway, transcription and pro-mRNA splicing in nucleus^27–33^. Extracellular monomer Galectin-3 could induce endothelial cell morphogenesis and angiogenesis in kinds of cancer^34^. Extracellular Galectin-3 is able to pentamerize. Pentamer Galectin-3 could crosslink glycosylated ligands to form a dynamic lattice on cell surface^35^. The lattice regulates the residing and endocytosis of plasma membrane glycoproteins and glycolipids. The lattice also regulates receptor kinase signaling and the functionality of membrane receptors, including the glucagon receptor, glucose and amino acid transporters, cadherins and integrin^36^. In addition, the lattice enhances tumour cell adhesion and migration in tumour progression^37^.

Cleavage of Galectin-3 by ﻿matrix metalloproteinase﻿s (MMPs) generates a single truncated Galectin-3 carbohydrate recognition domain (Gal3C), which binds to the glycoconjugate more tightly and could inhibit wound healing^38, 39^. In the bone remodelling process during skeletal metastasis of breast and prostate cancers, secreted intact Galectin-3 could induce osteoclastogenesis, whereas Gal3C could inhibit osteoclast differentiation, indicated that Gal3C may play an opposing role^40^. Gal3C inhibited tumour growth and metastasis in orthotopic nude mouse models of human breast cancer and in a murine model of human multiple myeloma^41, 42^.

Proteomics is a powerful tool to reveal the mechanism of diseases on the proteomics level. Stable Isotope Labelling with Amino acids in Cell culture (SILAC)^43^ provides an excellent quantitative proteomics approach for identifying dynamic alterations in protein expression and modification profiles under different stimuli^44^. Recently, quantitative proteomics approaches based on SILAC, liquid chromatography-mass spectrometry (LC-MS) and bioinformatics analysis have been widely applied in exploring mechanisms of tumourigenesis, metastasis and biomarker discovery of HCC^45–48^.

Although some research has been done to explore the anti-cancer or carcinogenic function of Gal3C in different types of cells, its potential anti-tumour mechanism in HCC is still unclear. Considering that Gal3C contains the same ligand binding site as Galectin-3, it may competitively bind the same ligands as Galectin-3 and disrupt Galectin-3-mediated processes in the extracellular space to provide an anti-Galectin-3 therapy. Here, for the first time, we report effects of Gal3C on HCC and its possible mechanism. Here, for the first time, we report effects of Gal3C on HCC and its possible mechanism. A recombinant carbohydrate recognition domain of Galectin-3 (rGal3C) was used to stimulate cells from the HepG2 HCC cell line. Changes in protein expression profiles were identified by quantitative proteomics based on SILAC and LC-MS/MS. Gene Ontology (GO), disease association and protein-protein interaction analysis found a series of carcinoma-related proteins affected by rGal3C. Immunoblotting and immunofluorescence results revealed that the possible anti-tumour mechanism of rGal3C in HCC involved the FAK/SRC pathway.

## Results

### rGal3C binds to cell surface of HepG2 and negatively affects viability, migration and invasion of HepG2

**Figure 1:**
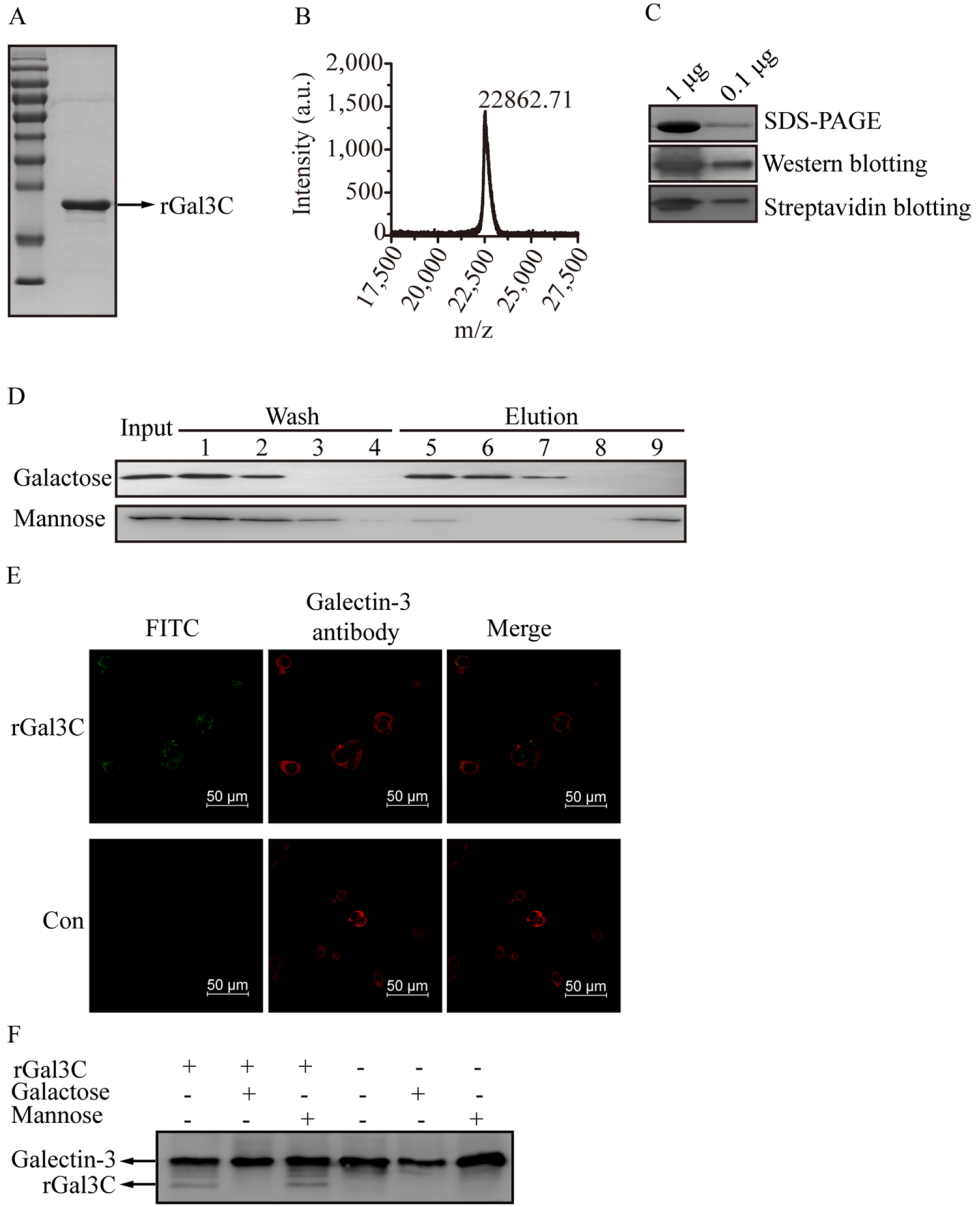
rGal3C is able to bind immobilized lactose and HepG2. (**A**) The 22.86 kDa band of rGal3C was detected. (**B**) Molecular weight of rGal3C detected by MALDI-TOF MS is 22862.71, which is consistent with its theoretical molecular weight. (**C**) Biotinylation of rGal3C was detected. (**D**) Binding of rGal3C to immobilize lactose was identified. Input fraction, washing fractions (band 1–4), eluting fractions (band 5–8) and NaOH eluting fraction (band 9) were detected. (**E**) Binding of rGal3C to cell surface of HepG2 was identified. Red fluorescence of Alexa 594 dye displayed total distribution of both Galectin-3 and rGal3C, while green fluorescence of FITC displayed only rGal3C binding to HepG2. (**F**) Galactose disrupted binding of rGal3C or Galectin-3 to HepG2. Cells washed with galactose displays a decreased Galectin-3 band, while mannose washing has no effect. HepG2 treated by rGal3C displays an rGal3C band which could be attenuated by galactose washing, but not mannose. Con, untreated HepG2 cells.

Expression of rGal3C was induced in BL21 (DE3, pET28a-AG/pBirA) with 0.1 mM IPTG at 30 °C for 8 hours. Then, the recombinant protein was purified by Ni-NTA agarose affinity chromatography and detected by SDS-PAGE (Fig. 1A and Supplementary Fig. S1). The molecular mass detected by matrix assisted laser desorption ionization-time of flight mass spectrometry (MALDI-TOF MS) was 22.86 kDa (Fig. 1B), which is consistent with its theoretical molecular weight. Biotinylation was confirmed by streptavidin-biotin blotting (Fig. 1C). There was an obvious binding and residing of rGal3C on the lactose agarose, and binding could be dissociated by 100 mM galactose, while mannose can hardly elute rGal3C from the lactose agarose, indicating that rGal3C possessed galactose binding activity (Fig. 1D). Binding of rGal3C on the HepG2 cell surface was identified by immunofluorescence and Western blotting. Compared with control, rGal3C binding on HepG2 cell surfaces displayed obvious green fluorescence from FITC. Total extracellular galectin-3 and rGal3C binding on the surface of HepG2 cells displayed red fluorescence from Alexa 594, revealing cell morphology (Fig. 1E). Compared with control cells, rGal3C-treated HepG2 cells displayed an obvious rGal3C band, and galactose but not mannose washing could decrease this band (Fig. 1F). Galactose but not mannose washing could also decrease the galectin-3 band, possibly because extracellular galectin-3 was washed away. These results suggested that rGal3C could bind to the HepG2 cell surface due to its galactose binding activity.

**Figure 2:**
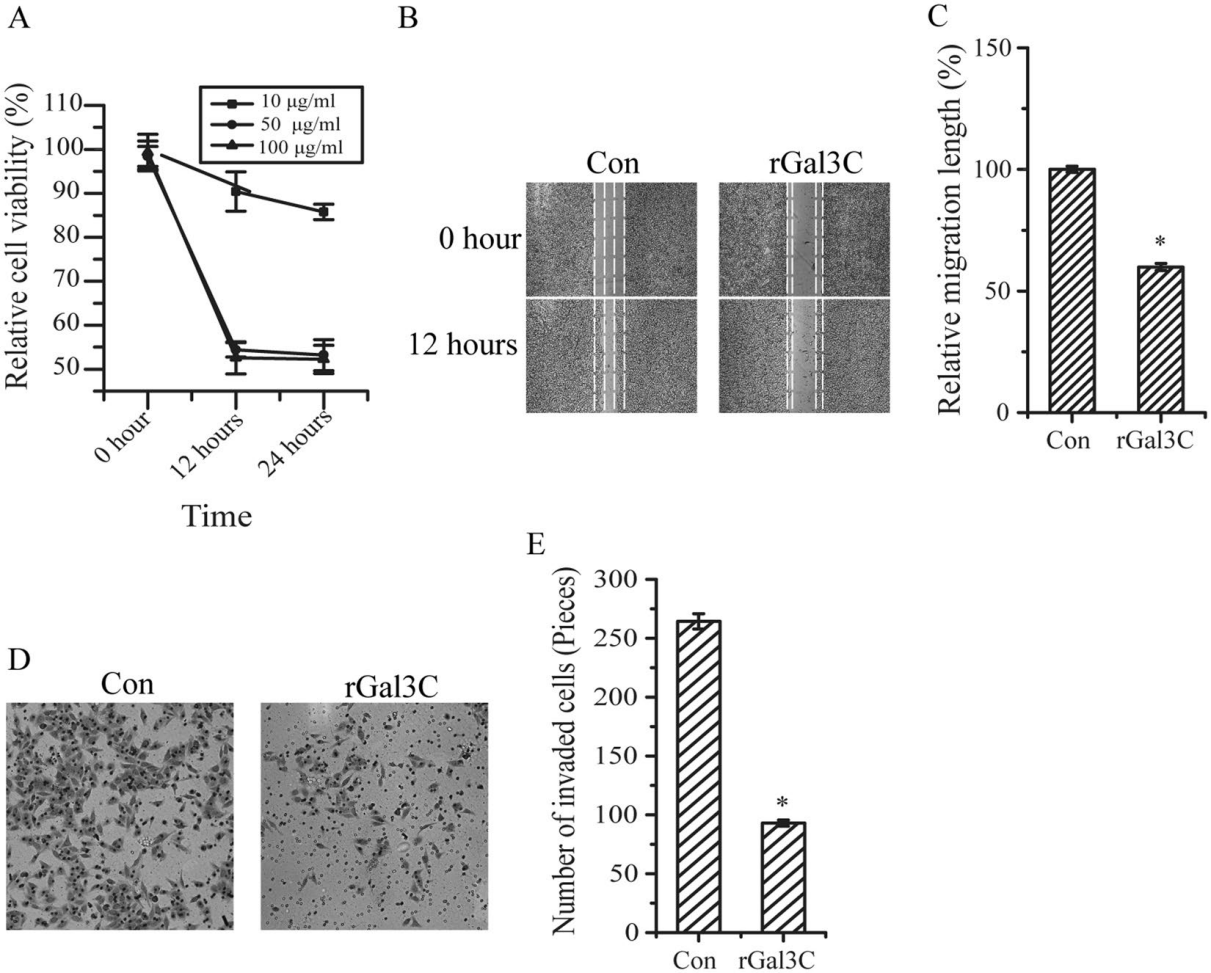
rGal3C negatively affects viability, migration and invasion of HepG2 cells. (**A**) The viability of HepG2 cells was inhibited after treated by 10, 50 and 100 μg/mL of rGal3C for 12 or 24 hours. (**B**) Cell migration was attenuated after stimulated by 50 μg/mL rGal3C for 12 hours. (**C**) Relative cell migration was attenuated to 59.8 ± 1.5% in HepG2 cells treated by 50 μg/mL rGal3C for 12 hours. Representative images were shown on the left, and the quantification of three replicates was shown. (**D**) The invasive capacity of HepG2 cells was inhibited after stimulated by by 50 μg/mL rGal3C for 12 hours. (**E**) The invaded cell munber was decreased (from 264 ± 6 to 93 ± 3) after stimulated by 50 μg/mL rGal3C for 12 hours. The quantification of three replicates was shown. Error bars indicate the mean ± SD. *p < 0.01. Con, untreated HepG2 cells.

Effects of rGal3C on the viability, migration and invasion of HepG2 cells were determined. Compared with control cells, cells stimulated with rGal3C displayed a significant decrease in cell viability (Fig. 2A). With increasing treatment time and concentration, the inhibition effect became more obvious. We found that HepG2 treated with 50 or 100 μg/mL of rGal3C for 12 or 24 hours displayed similar attenuation of cell viability (54.4 ± 1.6% for 50 μg/mL, 12 hours; 53.2 ± 3.5% for 50 μg/mL, 24 hours; 52.6 ± 3.6% for 100 μg/mL, 12 hours; 52.2 ± 3.2% for 100 μg/mL, 24 hours; n = 3), thus we decided to use 50 μg/mL of rGal3C to treat HepG2 for 12 hours in next experiments.

Compared with rGal3C-treated cells, untreated cells displayed remarkable migration (Fig. 2B and C). The migration of HepG2 cells was attenuated (59.8 ± 1.5%, n = 3) under the continuous influence of rGal3C. HepG2 cells treated with rGal3C displayed decreased invasion, indicating that the cell invasion ability was attenuated (Fig. 2D and E). These results suggested that rGal3C could negatively affect HepG2 cell invasion and migration.

### Protein identification and quantification using SILAC

**Figure 3:**
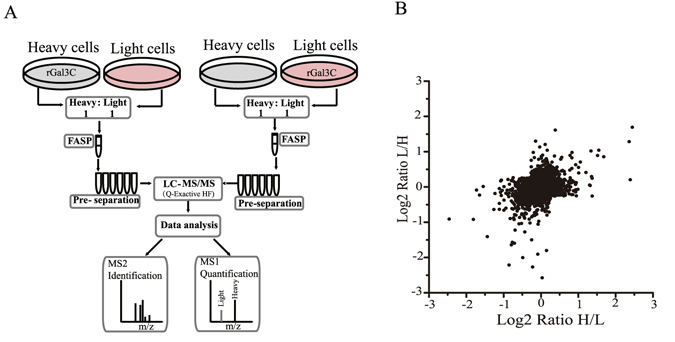
Quantitative proteomics analysis revealed the differentially expressed proteins after rGal3C treatment. (**A**) General workflow of the SILAC experiment. (**B**) Distribution of SILAC ratio of both experiments. Compared with untreated group, 80 proteins were up-regulated (log2 [ratio] > 0.58) and 110 were down-regulated (log2 [ratio] < −0.58).

The rGal3C-stimulated cells and control cells were subjected to quantitative proteomics analysis using the Stable Isotope Labelling with Amino acids in Cell culture (SILAC) technique to obtain an in-depth view of the proteomic regulation upon rGal3C stimulating. General workflow of the quantitative proteomics approach based on SILAC and LC-MS/MS is shown in Fig. 3A. HepG2 cells were cultured with DMEM containing ^13^C_6_-L-lysine and ^13^C_6_-L-arginine for at least 21 days to achieve a satisfactory labelling efficiency (Supplementary Fig. S2).

In total, 4705 and 4435 proteins were identified in the labelled and reversed-labelled experiments, respectively, of which 3945 overlapping proteins were identified and 3611 proteins were quantified. Proteins with SILAC ratio greater than 1.5 or less than 0.67 in either experiment were considered differentially regulated proteins. Under this cutoff value, 80 up-regulated proteins and 110 down-regulated proteins were identified (Table 1 and Supplementary Tables S1 and S2). The SILAC ratio distribution of the quantified proteins is shown in Fig. 3B. Eleven up-regulated proteins and 24 down-regulated proteins in both experiments were considered highly reliable, and their ratio and unique peptide number are shown in Table 2.

**Table 1:** The numbers of protein identified and quantified using SILAC.

	Experiment	Reverse experiment	Overlap proteins	Quantified proteins	Up-regutaed proteins (Ratio >1.5)	Down-regulated proteins (Ratio <0.67)
Total identifications	4705	4435	3945	3611	80	110

**Table 2:** Information of up or down-regulated proteins in both experiments.

	Protein IDs	Gene names	Ratio		Unique peptides	
			H/L	L/H	H/L	L/H
Up-regulated proteins	Q14764	MVP	2.54	2.03	8	5
	P01011	SERPINA3	3.21	1.81	6	6
	P09382	Galectin-1	2.87	1.85	4	4
	Q13509	TUBB3	2.01	1.46	4	4
	Q99519	NEU1	1.91	1.54	4	3
	Q6PUV4	CPLX2	2.93	2.05	4	2
	Q9BQL6	FERMT1	1.54	1.86	3	3
	P37802	TAGLN2	1.78	1.97	3	2
	P52943	CRIP2	1.47	1.79	3	2
	P31949	S100A11	5.16	2.43	2	2
	O43301	HSPA12A	1.78	1.77	2	2
Down-regulated proteins	P04004	VTN	0.18	0.53	9	5
	P10909	CLU	0.29	0.53	11	6
	Q92597	NDRG1	0.37	0.38	9	9
	P02671	FGA	0.42	0.64	9	8
	O00584	RNASET2	0.46	0.64	7	9
	Q02338	BDH1	0.50	0.63	8	7
	P13674	P4HA1	0.52	0.66	15	17
	Q08AH3	ACSM2A	0.55	0.22	2	3
	Q16877	PFKFB4	0.57	0.54	5	4
	P08833	IGFBP1	0.58	0.32	2	4
	P10646	TFPI	0.58	0.65	6	6
	P05121	SERPINE1	0.59	0.34	9	12
	Q9C0E8	KIAA1715	0.60	0.65	7	4
	Q4G0N4	NADKD1	0.60	0.54	6	5
	P02787	TF	0.61	0.53	29	28
	P07148	FABP1	0.61	0.58	9	8
	P08319	ADH4	0.62	0.43	11	9
	Q02252	ALDH6A1	0.62	0.66	16	16
	Q9Y646	CPQ	0.63	0.57	4	3
	Q9UN36	NDRG2	0.63	0.66	7	5
	P09972	ALDOC	0.64	0.62	9	10
	P28332	ADH6	0.65	0.69	9	10
	Q96A26	FAM162A	0.66	0.65	6	4
	Q6UWM9	UGT2A3	0.67	0.48	8	6

### Data mining of differentially expressed proteins

**Figure 4:**
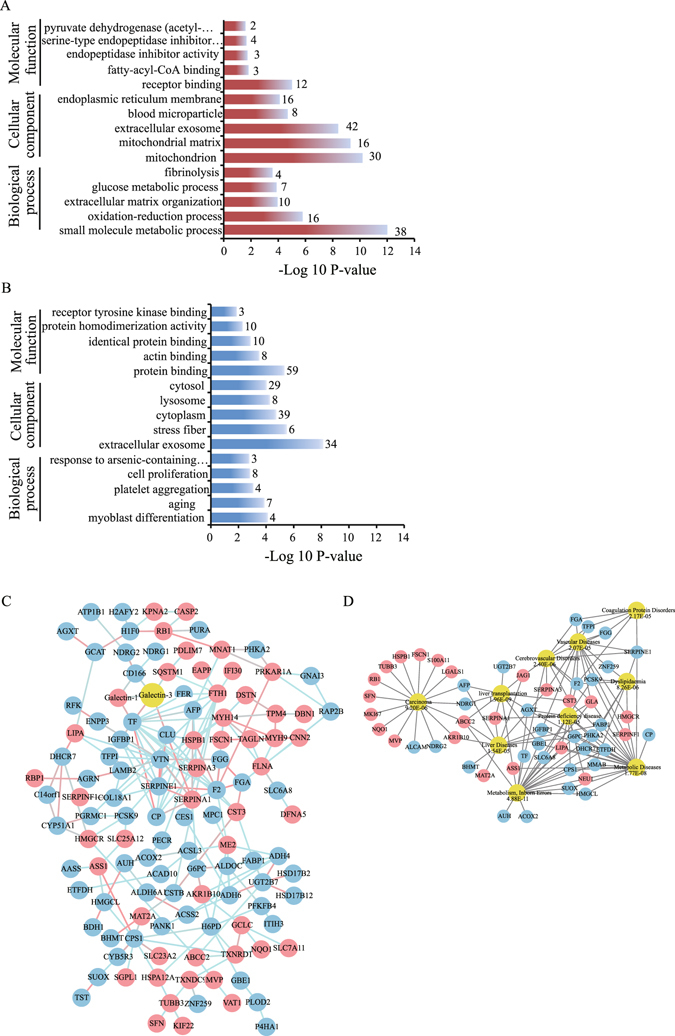
Gene Ontology, protein-protein interaction and disease association analysis of differentially expressed proteins upon rGal3C treatment. (**A**) Top five of biological process, cell component and molecular function of up-regulated proteins. (**B**) Top five of biological process, cell component and molecular function of down-regulated proteins. The −log^10^ p-value of the enrichment is shown on the x-axis, and the numbers represent the number of associated proteins associated with each term. (**C**) An interaction network of the proteins affected by rGal3C. The up-regulated proteins are represented by red nodes. The down-regulated proteins are represented by blue nodes and galectin-3 by yellow nodes. The red edge represents a strong close relationship between two nodes, and the blue edge represents an ordinary one. (**D**) Diseases associated with the differentially expressed proteins. The up-regulated proteins are represented by red nodes, and the down-regulated proteins are represented by blue nodes. Top ten of diseases associated with the differentially expressed proteins are represented by yellow ovals, and their P values are shown.

To obtain a biological view of 190 differentially expressed proteins, Gene Ontology (GO) enrichment analysis for biological process, cell component and molecular function was performed using DAVID Bioinformatics Resources 6.8^49, 50^. Most of the up-regulated proteins located in extracellular exosome, vesicles, adherent junctions and anchoring junctions, and involved in cell-cell adhesion and metabolism. Most of the down-regulated proteins located in mitochondria and extracellular exosome, and involved in processes of metabolism and oxidation-reduction processes (Fig. 4A and B).

Considering that rGal3C is the carbohydrate recognition domain of Galectin-3, protein-protein interactions of differentially expressed proteins with Galectin-3 were analysed by String 10^51^ (Fig. 4C). The analysis showed that TF, CD166, Galectin-1, CLU and SQSTM1 were related to Galectin-3.

To reveal the relationship between differentially expressed proteins and diseases, disease-association analysis of differentially expressed proteins was processed by WebGestalt^52, 53^. According to the analysis results (Fig. 4D), differentially expressed proteins were mainly separated into two main groups, one was related to carcinoma (9.20E-06), liver diseases (1.54E-05) and liver transplantation (1.96E-09), while the other was related to metabolic diseases (1.77E-08), inborn errors (4.18E-11), protein deficiency diseases (1.12E-05), cerebrovascular diseases (2.40E-06), vascular diseases (2.40E-06), dyslipidaemia (8.26E-06) and coagulation protein disorders (2.17E-05). These results suggested that rGal3C mainly acted on HepG2 tumour-related processes and substance metabolism.

**Figure 5:**
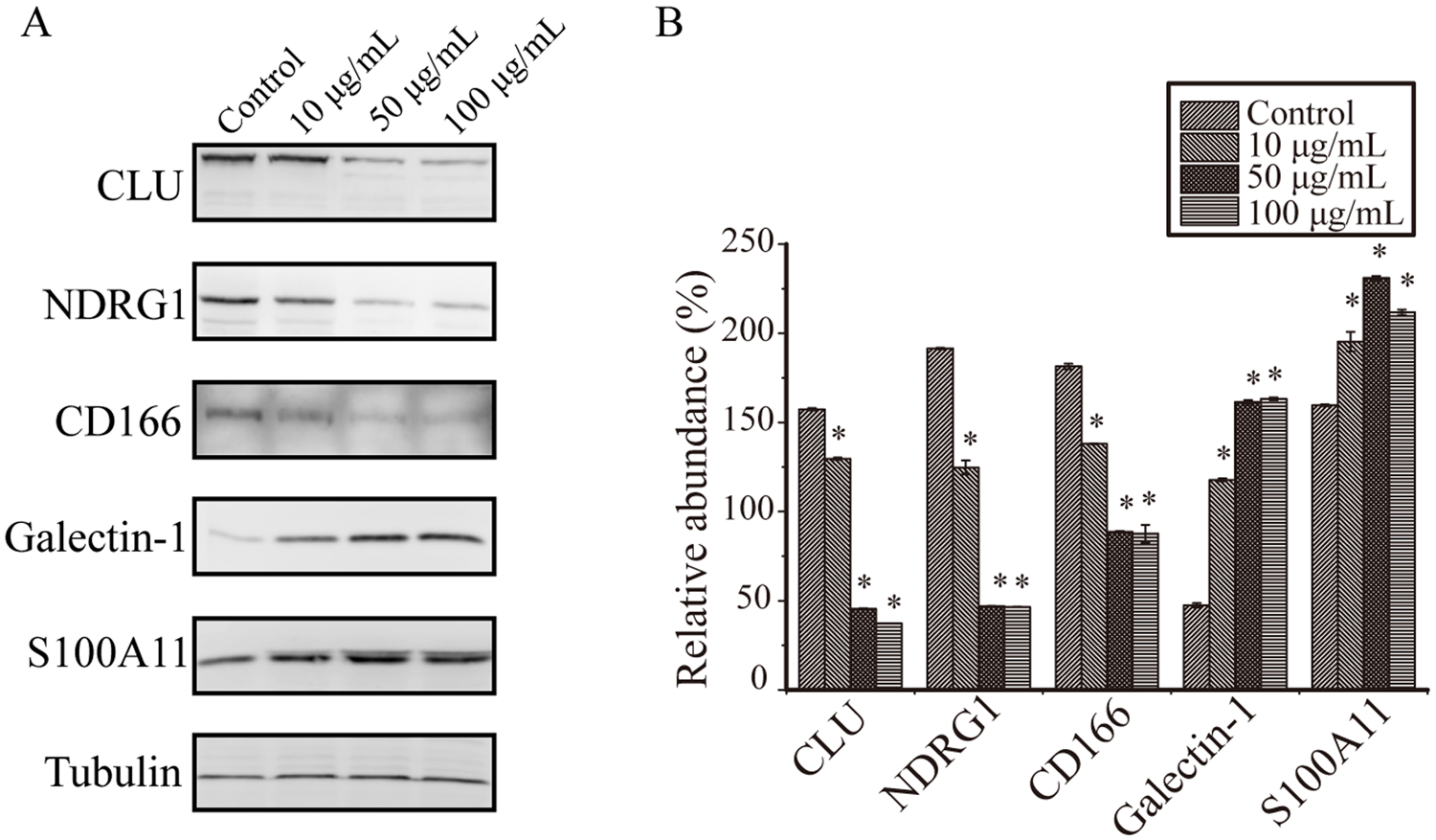
Dose-response western blotting verification of differentially expressed proteins. (**A**) The differential expression of CLU, NDRG1, ALCAM, Galectin-1 and S100A11 were displayed. (**B**) The western blot was quantitatively analyzed using Image J, and protein expression abundance was normalized to tubulin. Parallel experiments were progressed and representative results of three independent replicates were shown. Error bars indicate the mean ± SD. Control: untreated HepG2 cells; *p < 0.01.

Of these disease-associated proteins and proteins related to Galectin-3, NDRG1 showed a close relationship to carcinoma and liver diseases, and CD166, S100A11 and Galectin-1 were also related to carcinoma. Based on quantitative proteomics results and their potential metastasis-related function, differential expression of CLU, NDRG1, CD166, Galectin-1 and S100A11 were further validated by a dose-response western blotting (Fig. 5A and B). Compared with non-stimulated HepG2, S100A11 and Galectin-1 showed significant up-regulation, while NDRG1, CD166 and CLU showed significant down-regulation, and alterations were correlated with the concentration of rGal3C. These results were consistent with the results of SILAC-based quantitative proteomics.

### Potential receptors of rGal3C on cell surface of HepG2

To capture the potential receptors of rGal3C binding on cell surface of HepG2, ultraviolet radiation dependent cross-link strategy was employed. Binding of photo-Leucine analogue labelled rGal3C to HepG2 cell surface was displayed by fluorescein isothiocyanate (FITC). There was a remarkable green fluorescent spreading around the HepG2 cell surface after photo-leucine analogue labelled rGal3C incubation and ultraviolet radiation (Supplementary Fig. S3A and B).

**Figure 6:**
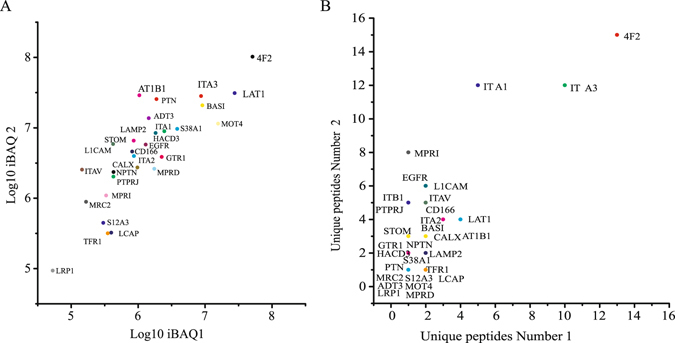
Label-free quantitative proteomics information of potential transmembrane receptors of rGal3C. (**A**) The distribution of iBAQ value in two independent repeated experiments. (**B**) The distribution of unique peptide number in two independent repeated experiments.

The released supernatant fraction enriched by streptavidin agarose was digested by FASP, and the peptide mixtures were identified by LC-MS/MS. Overall 177 proteins were identified in two independent repeated experiments, of which 30 proteins were predicted as transmembrane proteins by TMHMM Server v2.0 (Supplementary Fig. S3C and D and Supplementary Table S3). According to the label-free quantitative proteomics results (iBAQ value and unique peptide number) identified in two independent repeated experiments, we found that five integrins (ITA1, ITA2, ITA3, ITAV and ITB1) were enriched in 30 potential receptors of rGal3C on cell surface of HepG2, of which ITA3 was the most significant integrin, indicated that there may be interactions between rGal3C and ITA3 (Fig. 6A and B).

### rGal3C induces decrease of NDRG1 through the integrin/FAK/SRC pathway

**Figure 7:**
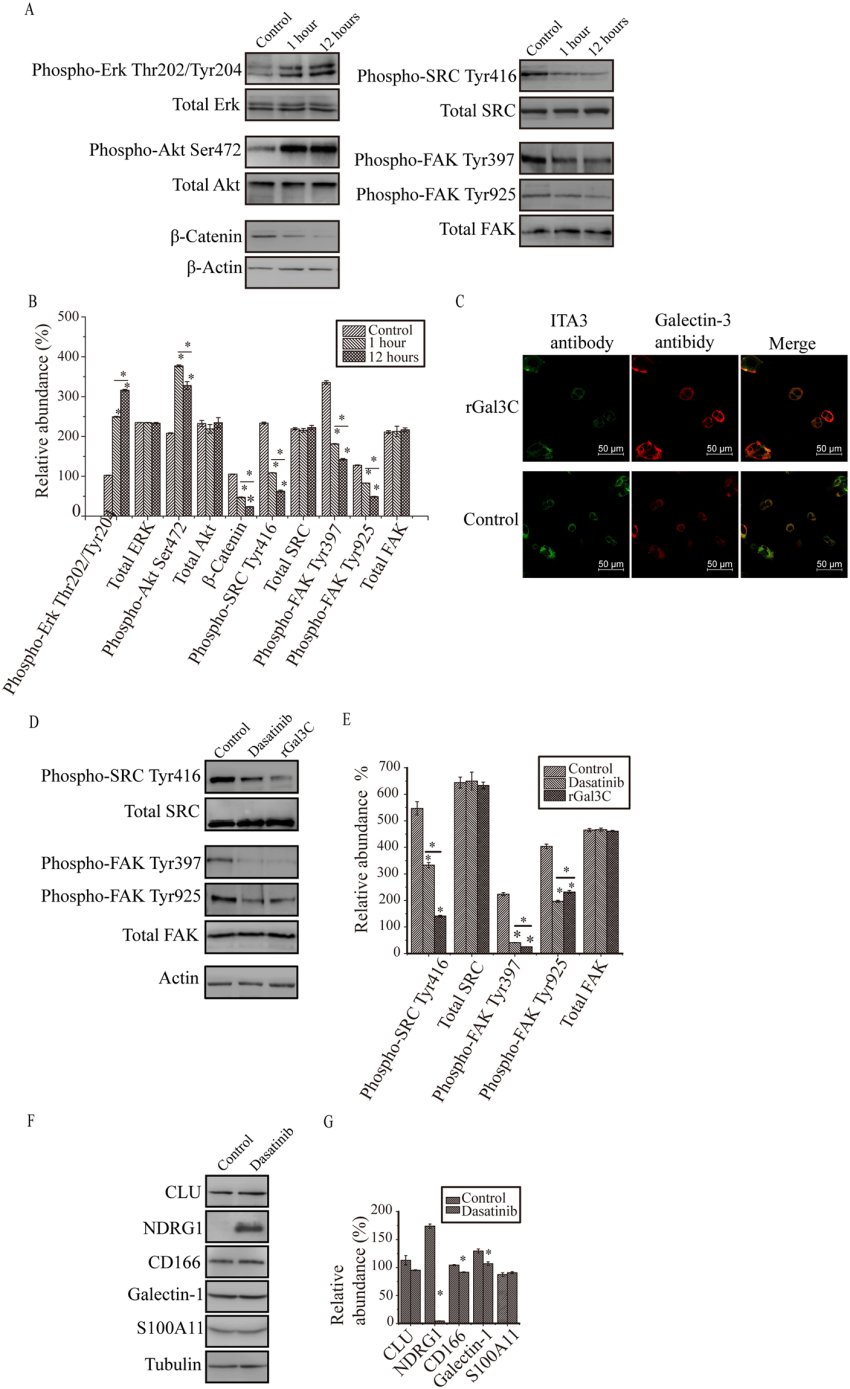
rGal3C regulates phosphorylation of FAK/SRC, integrin clustering and expression of proteins. (**A**) Phosphorylation of Erk1/2 at Thr202/Tyr204 and Akt at Ser472 were increased. Phosphorylation of FAK at Tyr397/Tyr925 and SRC at Tyr416 were suppressed, meanwhile expression of β-Catenin were decreased. Parallel experiments were progressed and representative results of three independent replicates were shown. (**B**) Relative abundance of β-Catenin and phosphorylated of Erk1/2, Akt, FAK/SRC and β-Catenin after rGal3C stimulating. Protein expression abundance was normalized to Actin. Results are representative of three independent experiments. (**C**) The total fluorescence of both galectin-3 and rGal3C of HepG2 cells treated by rGal3C was displayed by red fluorescence of Alexa 594 dye, while integrin clustering by green fluorescence of Alexa 488 dye. Normal HepG2 cells were used as control. (**D**) Both rGal3C and Dasatinib stimulating suppresses the phosphorylation of FAK at Tyr397/925 and SRC at Tyr416. Parallel experiments were progressed and representative results of three independent replicates were shown. (**E**) Relative abundance of phosphorylated FAK/SRC after rGal3C or Dasatinib stimulating. Protein expression abundance was normalized to Actin. Results are representative of three independent experiments. (**F**) Blocking of integrin/FAK/SRC signaling pathway by Dasatinib leads to the decrease of S100A11, Galectin-1, NDRG1, CD166 and CLU. Parallel experiments were progressed and representative results of three independent replicates were shown. (**G**) Relative abundance of CLU, NDRG1, CD166, Galectin-1 and S100A11 after Dasatinib stimulating. Protein expression abundance was normalized to tubulin. Results are representative of three independent experiments. Error bars indicate the mean ± SD. Con, untreated HepG2 cells; *p < 0.01.

To explore the pathway by which rGal3C affects HepG2 cells, alteration of phosphorylation of Erk1/2, Akt and FAK/SRC were detected by western blotting (Fig. 7A and B). Increased phosphorylation of Erk1/2 and Akt were identified, while reduced phosphorylation of SRC at Tyr416 and FAK at Tyr397 and Tyr 925 were identified. These results suggested that Integrin/FAK/SRC pathway was inhibited after rGal3C stimulating.

Integrin clustering on the cell surface is important for activation of the FAK/SRC pathway. Our former result indicated the possible interaction between rGal3C and integrin (ITA1, ITA2, ITA3, ITAV and ITB1). Thus, integrin distribution on the surface of rGal3C-stimulated HepG2 and control cells was identified by immunofluorescence with confocal microscopy. Compared with control cells, cells stimulated with rGal3C displayed less fluorescent foci formed by integrin, indicated that integrin clustering might be partially disrupted after rGal3C stimulation (Fig. 7C).

We then investigated whether integrin/FAK/SRC pathway played a role in rGal3C regulated proteins. Dasatinib, a specific inhibitor of SRC in HepG2, was used to stimulate HepG2 cells for 12 hours at a concentration of 10 nM. Dasatinib treatment led to a remarkable decrease of phosphorylation in SRC at Tyr416 and FAK at Tyr397 and Tyr 925, as well as rGal3C did (Fig. 7D and E). NDRG1 displayed the most significant alteration after Dasatinib stimulation, suggested that NDRG1 was a main signature molecules regulated by rGal3C through the integrin/FAK/SRC signalling pathway (Fig. 7F and G).

## Discussion

Liver cancer is one of the major health problems worldwide, especially in developing countries. Hepatocellular carcinoma (HCC) is the most common malignant liver cancer, and it has the highest cancer mortality rate, with increasing incidence in the modern world. During 2012, it is estimated that 782,500 new liver cancer patients and 745,500 deaths occurred worldwide, and 50% of the total number were in China^1^. Galectin-3, a mammalian lectin who could bind N-acetyllactosamine, is up-regulated in HCC and considered as a possible prognostic marker of HCC^37^. It could promote tumour progression based on its various functions in the cytoplasm, nucleus and extracellular microenvironment^54^. Thus, anti-Galectin-3 therapy may be an effective method for cancer treatment. Gal3C is the truncated carbohydrate recognition domain of Galectin-3, which contains the same ligand binding site as Galectin-3, but could not form pentamer, it has been reported that Gal3C is a potentially new molecule for treating breast cancer^41^, multiple myeloma^42^ and ovarian cancer^55^. Considering that Gal3C contains the same ligand binding site as Galectin-3, it may disrupt Galectin-3-mediated pathways on the cell surface through competitively bind the same ligands as Galectin-3.

Although some research has been done to explore the anti-tumour function of Gal3C in different types of cells, its potential anti-tumour mechanism in HCC is still unclear. In this study, we aim to explore the signature molecules and related pathways of Gal3C affecting HCC with quantitative proteomics approach.

Here, for the first time, we reported the signature molecules and related pathways of rGal3C affecting HCC. A recombinant carbohydrate recognition domain of Galectin-3 (rGal3C) was used to stimulate the HCC cell line, HepG2. Changes in protein expression profiles were identified by quantitative proteomics based on SILAC and LC-MS/MS. GO, disease association and protein-protein interaction analysis of differentially expressed proteins found a series of carcinoma-related proteins affected by rGal3C. Potential receptors of rGal3C were explored by an UV cross-linking capture strategy. Our results suggested the possible anti-tumour mechanism of rGal3C in HCC involved in Integrin/FAK/SRC pathway and NDRG1.

Firstly, we hope to prove that if Galectin-3 loses its N-terminal collagen domain and then could not form pentamer, it may loss its ability of promoting tumour procession, even inhibit cell growth and motility. We mainly focused on the effect of rGal3C on HepG2, thus HepG2 without rGal3C treatment was used as a suitable negative control in this study. rGal3C without N-terminal collagen domain was produced in *E. coli*, and its molecular weight detected by MALDI-TOF is 22.86 kDa (Fig. 1A and B and Supplementary Fig. S1). According to the reported structure of Gal3C^56, 57^, we consider that the N-terminus and C-terminus of the rGal3C would locate at the opposite side of its carbohydrate binding interface. According to our results, rGal3C retains its galactose binding characteristic (Fig. 1D–F).

Binding of rGal3C to HepG2 cells could inhibit cell viability, invasion and migration (Figs 1D–F and 2). HepG2 stimulated with rGal3C displayed a remarkable attenuation of cell viability, which positive correlated with the concentration of rGal3C and stimulating time (Fig. 2A). These results supported our former conjecture that Gal3C would inhibit cell growth and motility if it loses its oligomeric ability, suggested that rGal3C has the potential as an anti-tumour molecule in therapy of HCC.

Quantitative proteomics based on SILAC and LC-MS/MS offers a powerful tool to comprehensively discover the signature molecules of rGal3C-stimulated in HCC and explore the related pathway of anti-tumour mechanism of action (Fig. 3A). In this study, we identified 80 up-regulated proteins and 110 down-regulated proteins after rGal3C stimulating by quantitative proteomics approach (Table 1 and Supplementary Tables S1 and S2). GO analysis indicated that up-regulated proteins were located mainly in extracellular exosome/vesicle and involved in cell-cell adhesion and metabolism, and down-regulated proteins were located in mitochondria and extracellular exosome, and related to the processes of metabolism and oxidation-reduction (Fig. 4A and B). Protein-protein interaction analysis by String 10 indicated that the down-regulated proteins CD166, CLU and TF and the up-regulated proteins Galectin-1 and SQSTM1 were related rGal3C (Fig. 4C). Disease-association analysis results indicate that these differentially expressed proteins had a close relationship with carcinoma and metabolic disease (Fig. 4D).

Our quantitative proteomics results and subsequent bioinformatics analysis demonstrates a global view of rGal3C-stimulated HepG2 in protein expressing and biological process level. Extracellular rGal3C bound to HepG2 cell surface, then induced a series of changes in protein expression profile, and cell-cell adhesion, energy metabolism and exosome secretion were affected by rGal3C stimulating. These results suggest that rGal3C could regulate intercellular signature molecules and related pathways. CLU, NDRG1, CD166, S100A11 and Galectin-1 were associated with carcinoma and Galectin-3. Dose-response immunological experiment results displayed that their differential expression were correlated with the concentration of rGal3C, thus, they were possible signature molecules regulated by rGal3C (Fig. 5). These results were consistent with the results of SILAC-based quantitative proteomics.

NDRG1 is associated with carcinoma and liver transplantation, which strongly indicates that it might be closely related to HCC tumourigenesis and therapy. It has been reported that NDRG1 is a critical initiation factor for invasion of HepG2 cells^13^. In the present, the regulation of human NDRG1 gene expression has remained obscure, and limited studies have shown that the expression of NDRG1 is inhibited by Myc, and some chemical inducers such as NO, nickel (Ni^2+^) and cobalt (Co^2+^), as well as chemical iron chelators, can induce its expression^58–61^. Suppressed expression of NDRG1 significantly reduces cell proliferation and invasion in HepG2 cells and recovers senescence signaling^62, 63^. Overexpression of NDRG1 is often associated with tumour cell survival, significant portal vein invasion, intrahepatic metastasis, poor prognosis and shorter overall survival after liver transplantation^13, 64, 65^. These results strongly suggested that reducing intracellular NDRG1 levels is one possible therapeutic approach for HCC. In this study, we confirmed that rGal3C could effectively induce decrease of NDRG1 in HepG2, suggested that rGal3C could play its anti-tumour role through down-regulation of NDRG1 in HCC.

The related regulatory pathway of rGal3C towards NDRG1 in HCC is still unclear. Considering that rGal3C was located in extracellular space, it may process its regulatory role towards NDRG1 by binding receptors on HepG2 cell surface. It has been reported that extracellular Galectin-3 binds to a large number of membrane glycosylation ligands, affecting their distribution on the membrane and their signal transductionfunction^54^. Galectin-3 is able to pentamerize in the extracellular space and form lattice structures by crosslinking with its glycosylated receptors on the cell surface to regulate transmembrane signaling^36, 66^. Gal3C loses its oligomerization capacity, but gains higher ligand binding activity^67^, therefore rGal3C may competitive bind to the receptors of Galectin-3, and disrupt the lattice structure, resulting in negatively regulating the functions of extracellular Galectin-3. Thus, rGal3C may competitively inhibit Galectin-3 mediated transmembrane signaling through the same pathway.

In this study, 30 potential receptors of rGal3C on cell surface of HepG2 were identified by crosslinking and capturing rGal3C binding molecules using photo-leucine analogue labeled and biotinylated rGal3C (Supplementary Fig. S3 and Supplementary Table S3). We found that five integrin (ITA1, ITA2, ITA3, ITAV and ITB1) were enriched, of which ITA3 was one of the most significantly molecules according to their iBAQ value and unique peptide number (Fig. 6). These results suggested that integrin may be the main receptors of rGal3C on HepG2 cell surface. Integrin are glycoproteins on cell membrane which have been reported as receptors of Galectin-3, and Galectin-3-integrin interaction plays an important role in cell adhesion^36^. Galectin-3-integrin clustering could promote integrin activation as well as ligand-induced integrin activation^68, 69^. Integrin on membrane could regulate the activation of Erk, Akt and FAK/SRC pathway, thus the relationship between rGal3C stimulation and these pathways were identified.

Phosphorylation of Erk1/2, Akt and FAK/SRC were detected by western blotting. Decreased phosphorylation of FAK/SRC was identified in this study, while increased phosphorylation of Erk and Akt was also detected (Fig. 7A and B). These results are consistent with reported conclusions that Gal3C could induce Erk phosphorylation in HeLa cells^70^, and Galectin-3 is important for FAK/SRC signalling transduction^71^. The FAK/SRC pathway is always abnormally activated in HCC, and it contributes to invasion and metastasis, leading to poor survival^5–8^. These results suggested that decreased phosphorylation of FAK/SRC may be one main factor leading to decreased invasion and migration of HepG2 cells.

Auto-phosphorylation of FAK and activation of SRC is regulated by integrin clustering which dependents on Galectin-3 on the surface of cancer cells^72–77^. Auto-phosphorylation of FAK at Tyr397 induced by integrin clustering can create a high-affinity binding site for activated SRC, and activated SRC can trans-phosphorylate FAK at Tyr576/577, Tyr861 and Tyr925, thereby leading to the formation of a transient FAK/SRC signalling complex^78^. Therefore, a reasonable speculation is that rGal3C may induce decreased phosphorylation of FAK and SRC by disrupting Galectin-3-integrin clustering on the HepG2 cell surface. To confirm this inference, α3 integrin on the surface of HepG2 cells after rGal3C treatment were examined by immunofluorescence (Fig. 7C). Compared with control cells, rGal3C-stimulated HepG2 cells displayed less fluorescent foci, indicating that integrin clustering was partially disrupted. Thus, rGal3C can decrease integrin clustering and phosphorylation of FAK/SRC, thereby blocking FAK/SRC-mediated signalling.

We then determined that rGal3C-induced NDRG1 down-regulation was dependent on FAK/SRC phosphorylation by a signal blocking experiments using Dasatinib. Dasatinib is an ABL/SRC tyrosine kinase inhibitor that can specifically inhibit BCR-ABL and SRC family kinases^79^. Dasatinib inhibits phosphorylation of SRC, SFK/FAK and PI3K/PTEN/AKT but not MAPK/Erk and JAK/Stat pathways in HCC cells. In HepG2 cells, phosphorylation of SRC could be effectively inhibited by 10 μM Dasatinib^80^. Our results indicated that phosphorylation of both SRC at Tyr416 and FAK at Tyr397/925 were suppressed dramatically by either rGal3C or Dasatinib (Fig. 7D and E). Decreased phosphorylation of SRC by Dasatinib led to significant down-regulation of NDRG1 (Fig. 7F and G). These results suggest that NDRG1 is mainly SRC-regulated downstream signature molecule in HCC.

**Figure 8:**
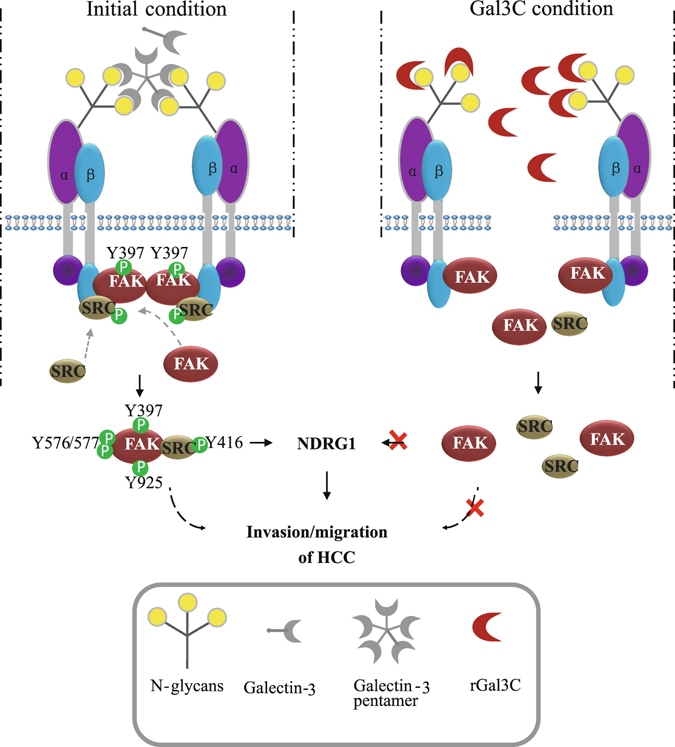
The diagram of rGal3C regulating NDRG1 through integrin/FAK/SRC pathway. rGal3C binds to the extracellular integrin, thereby resulting in the destruction of integrin clustering. Disintegrated integrin cannot induce phosphorylation of FAK/SRC to forming transient FAK-SRC signaling complex, and this may result in decreased expression of NDRG1 and attenuated migration and invasion of HCC.

According to our results, a possible mechanism by which rGal3C incudes dephosphorylating of FAK/SRC and further down-regulates NDRG1 and invasion and migration of HCC was identified. rGal3C could disrupt Galectin-3-integrin clustering on the surface of HepG2 cells, thereby leading to decreased phosphorylation of FAK and SRC. Inhibition of integrin/FAK/SRC signalling causes down-regulation of NDRG1, thereby resulting in decreased migration and invasion of HepG2 cells (Fig. 8). This is the first report of the possible mechanism by which extracellular Gal3C negatively regulates HCC invasion and migration. This study is an initial research of the anti-tumor mechanism of rGal3C to HCC, and revealing the global functions of rGal3C needs to be progressed in different HCC cell lines.

Exosomes are membrane-derived nanovesicles of about 30~100 nm released by most types of cells^81^. Cancer-derived exosomes have displayed multifunction in invasion and metastasis of HCC, and considered as diagnosis target and therapeutic tools^82^. Our results suggested that rGal3C may affect the composition of exosome secreted by HepG2. Although the detailed mechanism was unclear, this phenomenon provides us with a possible research direction that extracellular Galectin-3 may involve in exosome secretion of HCC.

Differential expression of CLU, CD166, S100A11 and Galectin-1 were also identified in this study (Fig. 5). CLU is a conserved glycoprotein that is involved in tissue remodelling, adhesion, apoptosis, proliferation, senescence and cell aggregation^83, 84^. CD166 is a potential biomarker, which acts as a cell adhesion molecule and is involved in metastasis, proliferation and anti-apoptosis in different types of cancers^14, 85, 86^. S100A11 is a calcium-binding protein that plays a role in the inhibition of cell proliferation in different types of cells^87–89^. In the HCC cell line JHH-5, S100A11 can inhibit DNA replication in the nucleus^90^. Galectin-1 is an overexpressed mammalian lectin that is associated with tumour-invasive characteristics and cell adhesion and polarization, as well as *in vivo* tumour growth and EMT^91–93^. Both Galectin-1 and Galectin-3 are located in the extracellular space and have similar functions, such as lattice formation and regulation of tumour adhesion, invasion and migration^94^. In this study, down-regulation of NDRG1, CLU and CD166 and up-regulation of S100A11 may all lead to decreased migration and invasion of HepG2 cells, while up-regulation of Galectin-1 may occur in a compensatory manner in response to the blocking of Galectin-3-regulated functions and keep the cells alive. Detail roles of these potential signature molecules in HCC still need further study.

In summary, for the first time, our study provides valuable insight into the anti-tumour mechanism of rGal3C involving integrin/FAK/SRC pathway and NDRG1 in HCC on a proteomics level. Our results suggest that rGal3C is an effective but not comprehensive anti-tumour molecule to HepG2 cells. The possible anti-tumour mechanism is that rGal3C disrupts Galectin-3-integrin clustering on the cell surface, resulting in suppression of integrin/FAK/SRC pathway and subsequent down-regulation of NDRG1 expression. rGal3C also induces up-regulation of S100A11 and Galectin-1, and down-regulation of CD166 and CLU, which can partially contribute to inhibition of HepG2 cell migration and invasion. Our results also suggest the possible relationship between extracellular Galectin-3 and exosome secretion of HepG2. This study provides useful guidance for developing therapies based on rGal3C to treat HCC, and more work is needed to reveal a more detailed mechanism of action for rGal3C regulation of HCC.

## Methods

### Protein expression

Plasmid pET28a-AG (Supplementary Figure S1) for rGal3C expression was synthesized (The Beijing Genomics Institute, China). pBirA was a plasmid expressing BirA in *E. coli* under IPTG inducing which was a gift of Doctor Kurt Drickamer^95^. BL21(DE3, pET28a-AG/pBirA) was cultured in LB medium to OD600 = 0.5, and then 10 μM IPTG and 10 μg/mL biotin were added to the culture to induce the expression of rGal3C at 30 °C, 120 rpm for 8 hours. The Origami2(DE3, pET28a-AG/pBirA) was cultured in M9 medium to OD = 0.3, and then 10 μM IPTG (Sigma-Aldrich, USA) with 10 μg/ml biotin (Sigma-Aldrich, USA), 25 μg/ml photo-reactive-leucine analogue (Pierce, USA) and 25 μg/ml leucine (Sangon, China) were added to into the medium at 30 °C, 120 rpm for 8 hours in the dark.

The bacterial sediment was collected and then broken in binding buffer (20 mM Tris-HCl, 300 mM NaCl, pH 8.0) by ultrasonic wave at 4 °C. The cell supernatant was extracted by centrifuged at 10000 g for 30 minutes at 4 °C. rGal3C was captured by a Ni-NTA agarose column (MCLAB, USA), and washed with cold his-tag washing buffer (20 mM Tris-HCl, 300 mM NaCl, pH 6.0) containing 100 mM imidazole. Then, rGal3C was eluted with 1 mL washing buffer containing 250 mM imidazole. This step was repeated for ten times. The eluted fractions were detected using 12% SDS-PAGE, dialyzed for 24 hours in PBS with 10% glycerol in 4 °C and then stored at −80 °C.

### Lactose binding assay

Ten μg of rGal3C was incubated with 100 μL of lactose-agarose (Sigma-Aldrich, USA) in 1 mL PBS in 1.5 mL column overnight at 4 °C. Lactose-agarose was washed with 1 mL of cold PBS for four times. Then, proteins bound to the lactose agarose were eluted with 1 mL PBS containing 100 mM galactose or 100 mM mannose for four times. The agarose was then washed with 1 mL 0.5N NaOH. Each fraction was collected. One fifth of each fraction was precipitated by incubation with 1 μg BSA and 800 μL acetone in 1.5 mL tubes at −20 °C. Tubes were centrifuged at 14000 g for 15 minutes at 4 °C, and the sediment was lyophilized and then detected by 12% SDS-PAGE and biotin-streptavidin blotting.

### Cell culture and Stable isotope labeling with amino acids in cell culture (SILAC) and protein sample preparation

HepG2 cells were cultured in DMEM (Hyclone, USA) containing 10% fetal calf serum (Gibco, USA), 100 units/mL penicillin and streptomycin at 37 °C with 5% carbon dioxide and 100% humidity. Then, cells were transferred to a cell culture medium containing 100 mg/mL of ^13^C_6_-L-lysine (Invitrogen, USA) and 100 mg/mL of ^13^C_6_-L-lysine (Invitrogen, USA). The cells were grown in heavy stable isotope reagent for at least 21 days^96^.

Heavy isotope amine acid labeled HepG2 cells or light cells were cultured for 12 hours, and then washed with cold sterile PBS (Gibco, USA) for three times. The cells were then stimulated with rGal3C.

Cells were washed and collected, and then dissolved in cell lysis buffer [100 mM Tris, 4% sodium dodecyl sulfonate (SDS), 1% protease inhibitor cocktail (Sigma-Aldrich, USA), pH 8.0] using ultrasonic waves. Then, the mixture was centrifuged at 14000 g for 15 minutes at room temperature. Cell lysis supernatant was collected and total protein concentration was measured by Nanodrop 2000C (Thermo Fisher Scientific, USA).

### Ultraviolet radiation dependent cross-link strategy for receptors capturing

HepG2 cells were cultured in 10 cm culture dishes (Corning, USA), and then washed PBS for three times. Then, 5 ml cold sterile PBS containing 250 μg photo-leucine analogue (Pierce, USA) labeled rGal3C was injected. Dishes were kept in dark for at least 4 hours at 4 °C, and then fixed with 30W, 365 nm UV for 15 minutes at a 5 cm distance. Fixed rGal3C were dtetcted by FTIC labelled streptavidin. Cells were collected and re-suspended by 1 mL PBS containing 0.5% triton X-100 and 1% protease inhibitor (Sigma-Aldrich, USA) and broken by ultrasonic wave at 4 °C. Cell supernatant was extracted, and captured fraction was enriched by 100 μL streptavidin agarose (Thermo Fisher Scientific, USA). Agarose was washed with 1M KCl and 0.1M Na_2_CO_3_, each for three times. Captured fraction released by heated at 95 °C for ten minutes in 100 μL 1% SDS solutionand then centrifuging at 10000 g for 15 minutes at room templature was loaded to FASP.

### Filter-aided sample preparation (FASP)

The protein digestion was processed by FASP with slight adaptations^97^. For SILAC experiment, treated and untreated samples were mixed in equal amount. Samples were denatured at 95 °C with 10 mM DTT for 10 minutes. Then, 50 mM iodoacetamide was added for 30 minutes at room temperature in dark. UA (50 mM Tris, 8 M Urea, pH 8.0) was added to the mixture to dilute the SDS to a final concentration of 0.1%. The mixture was transferred into a 500 mL/30 kDa ultra-fraction tube (Merck-Millipore, Germany). The ultra-fraction tube was washed with 200 μL UA and succedent 200 μL ABC (50 mM ammonium bicarbonate), each for three times by centrifuging at 14000 g for 15 minutes at room temperature. Then, 100 μL ABC containing 0.1 μg/μL trypsin (Progema, USA) was injected into each ultra-fraction tube. Tubes were incubated at 37 °C overnight. Peptides were collected by centrifuging at 14000 g for 15 minutes. Tubes were washed twice with 100 μL of ABC for 15 minutes. The flowing fractions were collected together, and the concentration was measured using a Nanodrop 2000C. Peptides were acidized with 10 μL 0.1% trifluoroacetic acid (TFA, Sigma-Aldrich, USA) and freeze-dried, and then stored at −80 °C.

### High pH reversed-phase prefractionation of the peptides

The peptides were pre-separated using high pH reversed-phase prefractionation. The pipet tip containing 5 mg of C18 reverse-phase material (Bonna-Agela Technologies, USA) was blocked by a C8 sieve plate. The tip was washed with 90 μL methanol, 90 μL acetonitrile, and then 90 μL alkaline water (pH = 10). Fifty μg peptides were re-dissolved in 160 μL of alkaline water (pH = 10) and injected into the tip. The tip was centrifuged at 1200 g for 5 minutes to remove the liquid. Then, 90 μL of alkaline water was injected into the tip, and then the tip was centrifuged at 1200 g for 5 minutes. Peptides were then eluted with increasing concentration (6%, 9%, 12%, 15%, 18%, 21%, 25%, 30%, 35%, 40%, 45% and 50%) of acetonitrile in alkaline water. Fractions were collected, and the last six fractions were mixed with the first six fractions. These six fractions were freeze-dried and stored at −80 °C.

### Liquid chromatography tandem mass spectrometry analysis of peptide mixture

The LC-MS/MS detecting system consisted of a nanoflow HPLC chromatographic instrument (Easynano LC 1200, Thermo Fisher Scientific, USA) coupled to a Q-Exactive HF mass spectrometer (Thermo Fisher Scientific, USA) with a Nano electrospray ion source (Thermo Fisher Scientific, USA) online. Briefly, 0.5 μg of peptide mixtures were transferred to the self-packed C18-reversed phase column (12 cm length, 150 μm inner diameter) using flowing phase A (99.9% water and 0.1% FA) and separated using a 78 min linear gradient of 6–40% flowing phase B (99.9% acetonitrile and 0.1% FA) at a flow rate of 600 nL/min. High-energy collisions induced dissociation mode and an Orbitrap mass analyzer were used in the analysis. The peptides were analyzed using data-dependent MS/MS acquisition with dynamic exclusion duration of 12 s. The resolution of MS1 was 120,000, and the maximum injection time was 80 ms. The resolution of MS2 was 15,000, and the maximum injection time was 45 ms, and the AGC target was 2E4. The scan range was 300–1,400 m/z for MS1 and 200–2,000 m/z for MS2. Twenty most intense precursor ions were selected for the MS/MS analysis.

### Database searching and data mining

Raw files were processed to Maxquant 1.5.3 to quantitatively information analyze protein expression^98^. The enzyme digestion mode was set as Trypsin/P and two missed cleavage sites. The fragmentation spectra were searched against the Swiss prot Human database containing 20193 proteins. The multipilcity was set as two for the samples labeled with for ^13^C_6_-L-lysine and ^13^C_6_-L-lysine. The variable modifications were set as oxidation (M), acetylation (protein N-terminal) and Deamidation (NQ). The fixed modification was set as carbamidomethyl (C). Unique and razor peptides were used for the quantitative analysis. All of the peptides were used, regardless of their modifications. The PSM FDR was set as 1% and protein FDR was set as 1%. The remaining parameters were set to the default modes.

The gene ontology and pathway analyses were conducted using DAVID Bioinformatics Resources 6.8 online (https://david.ncifcrf.gov/) for gene ontology(GO), String 10 (http://string-db.org/) for protein interaction analysis, WebGestalt (http://www.webgestalt.org)^53^ for Disease Association analysis and TMHMM v2.0 (http://www.cbs.dtu.dk/services/TMHMM/) were used for bioinformatics analysis.

### Cell viability, cell migration and invasion assay

For MTT assay, 10^4^ cells in each well were cultured in 96-well plates (Corning, USA), and 200 μL of DMEM containing 10, 50 or 100 μg/mL rGal3C was injected. After stimulating, 0.5 mg/mL MTT (Sangon, China) was injected into each well for incubating 4 hours. Then, each well was gently washed three times with cold sterile PBS, and 200 μL of DMSO (Sigma-Aldrich, USA) was injected into each well to dissolve the treated cells. The plate was shocked at 200 rpm for 10 minutes, and the absorbance at 490 nm of each well was measured. Each well was repeated for three times.

For wound-healing assay, cells were maintained in the 12-well plates (Corning, USA) to form confluent cell monolayer. Then, cells were incubated with 1 mL DMEM containing rGal3C. The confluent cell monolayer was wounded by manually scraping the cells with a 10 μL pipette tip. Then, 1 mL DMEM (Hyclone, USA) containing 50 μg/mL of rGal3C was injected. Migration was assessed after 12 hours. Wells without rGal3C stimulating were the control group.

The basement membrane invasion assay was performed using Transwell (Corning, USA). Generally, 10^4^ HepG2 cell was cultured in the insert coated with matrigel for 24 hours. Then, 600 μL DMEM containing 50 μg/mL of rGal3C and 10% FBS was added to the lower chamber, and 100 μL of DMEM containing 50 μg/mL of rGal3C was added into the insert. Cells were then incubated for 24 hours. The cells that invaded across and adhered on the bottom surface of the insert were fixed with 4% paraformaldehyde/PBS for 15 minutes at room temperature, then strained with 0.1% crystal violetand counted. There was no rGal3C in the control group.

### Immunofluorescence analysis

HepG2 cells were maintained in the confocal culture dishes, and treated with 50 μg/mL of rGal3C for 12 hours. Dishes were gently washed three times with cold sterile PBS, and fixed in PBS with 4% paraformaldehyde for 15 minutes at room temperature. Fixed cells were gently washed three times with cold sterile PBS, then 400 μL cold sterile PBS containing 2 μg anti-Galectin-3 rabbit polyclone antibody (Cell Signaling Technology, USA) was injected. Dishes were incubated in dark and moist box overnight at 4 °C, then gently washed three times with cold sterile PBS for three times. One μg Alexa 594 dye labeled anti-mouse IgG (Cwbiotech, China) and 1 μg FITC labeled streptavidin (Cwbiotech, China) in 400 μL PBS was added into each dish. Dishes were incubated in dark and moist box for 1 hour at 37 °C.

For displaying the distribution of integrin clustering on cell surface, anti-ITA3 mouse polyclone antibody (Cell Signaling Technology, USA) and anti-Galectin-3 rabbit polyclone antibody (Cell Signaling Technology, USA) were added the same as described above. The following steps were mostly the same expect using Alexa 488 dye labeled anti-mouse IgG (Cwbiotech, China) and Alexa 561 labeled anti-rabbit IgG (Cwbiotech, China).

Dishes were gently washed with cold sterile PBS for three times, and then placed under a confocal microscope (Nikon, Japan). Images were taken under 60-fold objective lens.

### Western blotting and biotin-streptavidin blotting

For the western blotting analysis, 30 μg of the total cell lysis was separated using 12% SDS-PAGE and transferred to a polyvinylidene fluoride membrane (Merck-Millipore, Germany) with a 0.22 μm aperture. After blocking with 5% bovine albumin dissolved in TBST (20 mM Tris-HCl, 150 mM NaCl, and 0.1% Tween-20, pH 8.0), the membrane was washed three times with TBST. Then, the membrane was incubated with primary antibody at a 1:1000 dilution overnight. The membrane was washed three times with TBST for three times, and then incubated with HRP conjugated secondary antibody at a 1:5000 dilution for two hours in room temperature. The membrane was washed three times with TBST, and then washed three times with TBS. The target protein bands were detected using ImageQuant LAS 500 (GE Healthcare, USA). The primary antibodies and secondary antibodies used in this study were purchased from Cell Signaling Technology (Cell Signaling Technology, USA).

For the biotin-streptavidin blotting analysis, the protein samples separating and transferring steps were the same as western blotting, but the membrane was incubated with HRP labeled streptavidin at a 1:500 dilution for at least 1 hour at room temperature. The membrane was then washed three times with TBST, and then washed three times with TBS. The target protein bands were detected using ImageQuant LAS 500 (GE Healthcare, USA).

### Statistical analysis

All tests were performed at least in triplicate, and all experiments were performed at least three times. The results are expressed as the mean ± standard deviation (SD). Differences between groups were analyzed using Student’s t-test or one-way analysis of variance (ANOVA). The analysis was conducted using Origin v9.0 software (OriginLab, USA), and differences were considered significant at *P ≤ 0.01.

## Electronic supplementary material

Supplementary information
