# Supplementary material for: Quantitative proteomics reveal the anti-tumour mechanism of the carbohydrate recognition domain of Galectin-3 in Hepatocellular carcinoma
Source: Sci Rep. 2017 Jul 12;7:5189. doi: 10.1038/s41598-017-05419-5 (PMC5507876; doi:10.1038/s41598-017-05419-5)
Supplement: Supplementary file 1 — Supplementary information [file 41598_2017_5419_MOESM1_ESM.pdf]

Quantitative proteomics reveal the anti-tumour mechanism of the carbohydrate recognition domain of Galectin-3 in Hepatocellular carcinoma

Mingchao Wang, Fang Tian, Wantao Ying*, Xiaohong Qian*

Correspondence to: QIAN Xiao hong, Email: qianxh1@163.com.YING Wan tao, Email: proteomics@126.com

Affiliations:

Beijing Institute of Radiation Medicine, National Center for Protein Sciences Beijing, State Key Laboratory of Proteomics, Beijing Proteome Research Center, Beijing, China

Tel/Fax: +86-10-80705055; +86-10-80705155

Running title: Anti-tumor mechanism revealed by quantitative proteomics

Key words: Hepatocellular carcinoma**,** Quantitative proteomics, Gal3C

A

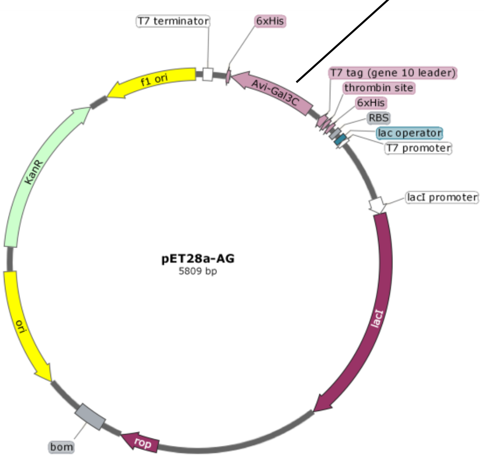

>Avi-Gal3C
ATGGCTGGGCCACTGATTGTGCCTTATAACCTGCCTTTG
CCTGGGGGAGTGGTGCCTCGCATGCTGATAACAATTCTG
GGCACGGTGAAGCCCAATGCAAACAGAATTGCTTTAGA
TTTCCAAAGAGGGAATGATGTTGCCTTCCACTTTAACCC
ACGCTTCAATGAGAACAACAGGAGAGTCATTGTTTGCAA
TACAAAGCTGGATAATAACTGGGGAAGGGAAGAAAGAC
AGTCGGTTTTCCCATTTGAAAGTGGGAAACCATTCAAAA
TACAAGTACTGGTTGAACCTGACCACTTCAAGGTTGCAG
TGAATGATGCTCACTTGTTGCAGTACAATCATCGGGTTA
AAAAACTCAATGAAATCAGCAAACTGGGAATTTCTGGT
GACATAGACCTCACCAGTGCTTCATATACCATGATAGGA
CTGAATGACATCTTCGAAGCACAGAAAATCGAGTGGCA
TGAG

B

>Sequencing result of Avi-Gal3C

ATGGGCAGCAGCCATCATCATCATCATCACAGCAGCGGCCTGGTGCCGCGCGGCAGCCATATGGCTAGCATGACT
GGTGGACAGCAAATGGGTCGCGGATCCGAATTCATGGCTGGGCCACTGATTGTGCCTTATAACCTGCCTTTGCCTG
GGGGAGTGGTGCCTCGCATGCTGATAACAATTCTGGGCACGGTGAAGCCCAATGCAAACAGAATTGCTTTAGATT
TCCAAAGAGGGAATGATGTTGCCTTCCACTTTAACCCACGCTTCAATGAGAACAACAGGAGAGTCATTGTTTGCAA
TACAAAGCTGGATAATAACTGGGGAAGGGAAGAAAGACAGTCGGTTTTCCCATTTGAAAGTGGGAAACCATTCAA
AATACAAGTACTGGTTGAACCTGACCACTTCAAGGTTGCAGTGAATGATGCTCACTTGTTGCAGTACAATCATCGG
GTTAAAAAACTCAATGAAATCAGCAAACTGGGAATTTCTGGTGACATAGACCTCACCAGTGCTTCATATACCATGA
TAGGACTGAATGACATCTTCGAAGCACAGAAAATCGAGTGGCATGAGCTCGAGCACCACCACCACCACCACTGA

C

>rGal3C

MGSSHHHHHHSSGLVPRGSHMASMTGGQQMGRGSEFMAGPLIVPYNLPLPGGVVPRMLITILGTVKPNANRIALDFQR
GNDVAFHFNPRFNENNRRVIVCNTKLDNNWGREERQSVFPFESGKPFKIQVLVEPDHFKVAVNDAHLLQYNHRVKKL
NEISKLGISGDIDLTSASYTMIGLNDIFEAQKIEWHELEHHHHHH

Supplementary Figure S1. Informtion of pET28a-AG and rGal3C.(A) Plasmid pET28a-AG was synthesized by inserting the 468 bp Avi-Gal3C DNA fragment into plasmid pET28a. (B) Nucleotide sequencing results of the inserted DNA fragment(Avi-Gal3C), the DNA fragment encoding carbohydrate recognize domain of Galectin-3 was underlined. (C) Amino acid sequence of rGal3C. The first 36 amine acids were the N-terminal histione tag (HisHisHisHisHisHis) and T7 tag(MetAlaSerMetThrGlyGlyGlnGlnMetGly), and underlined amine acids from 37-177 were the core sequence of carbohydrate recognition domain of galectin-3, and amine acids from 178-200 were the avi-tag(GlyLeuAsnAspIlePheGluAlaGlnLysIleClu) and C-terminal histione tag (HisHisHisHisHisHis).

IPAMTIA**K**

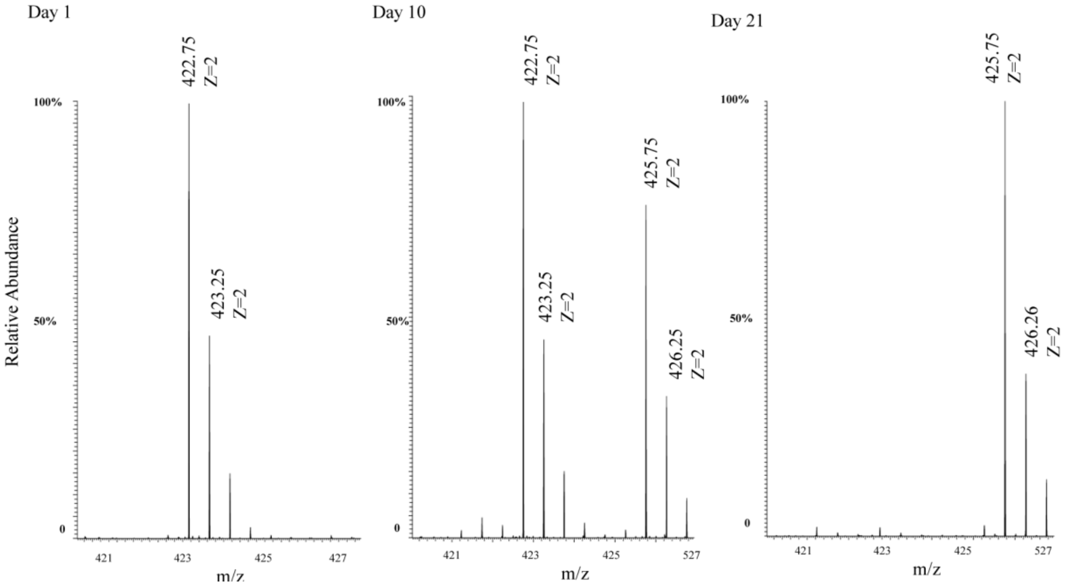

LLSELDQQSTEMP**R**

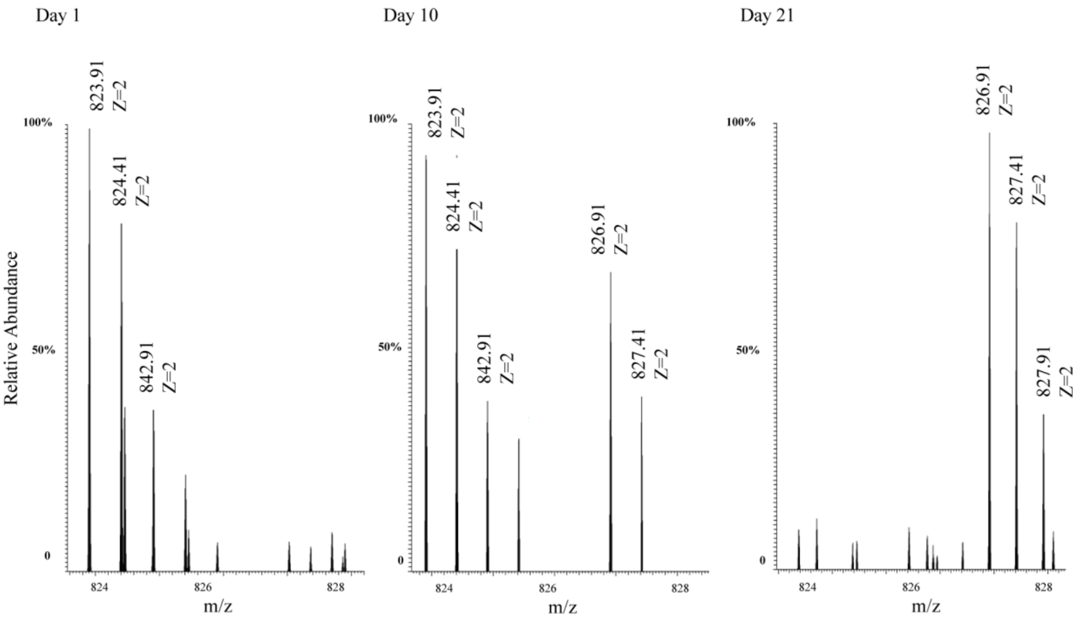

Supplementary Figure S2. Incorporation of $^{13}C_6$-L-lysine and $^{13}C_6$-L-arginine into HepG2 at various time points. The peptide IPAMTIAK (Mr=843.49) has a charge state of 2 and contains one lysine, the peptide LLSELDQQSTEMPR (Mr=1645.84) has a charge state of 2 and contains one arginine, and their heavy isotope is 6.032 Da higher than the light isotope. The ratio of intensity of heavy to light peak steadily increased. On day 21, this peptide was fully incorporated with $^{13}C_6$-L-lysine or $^{13}C_6$-L-arginine.

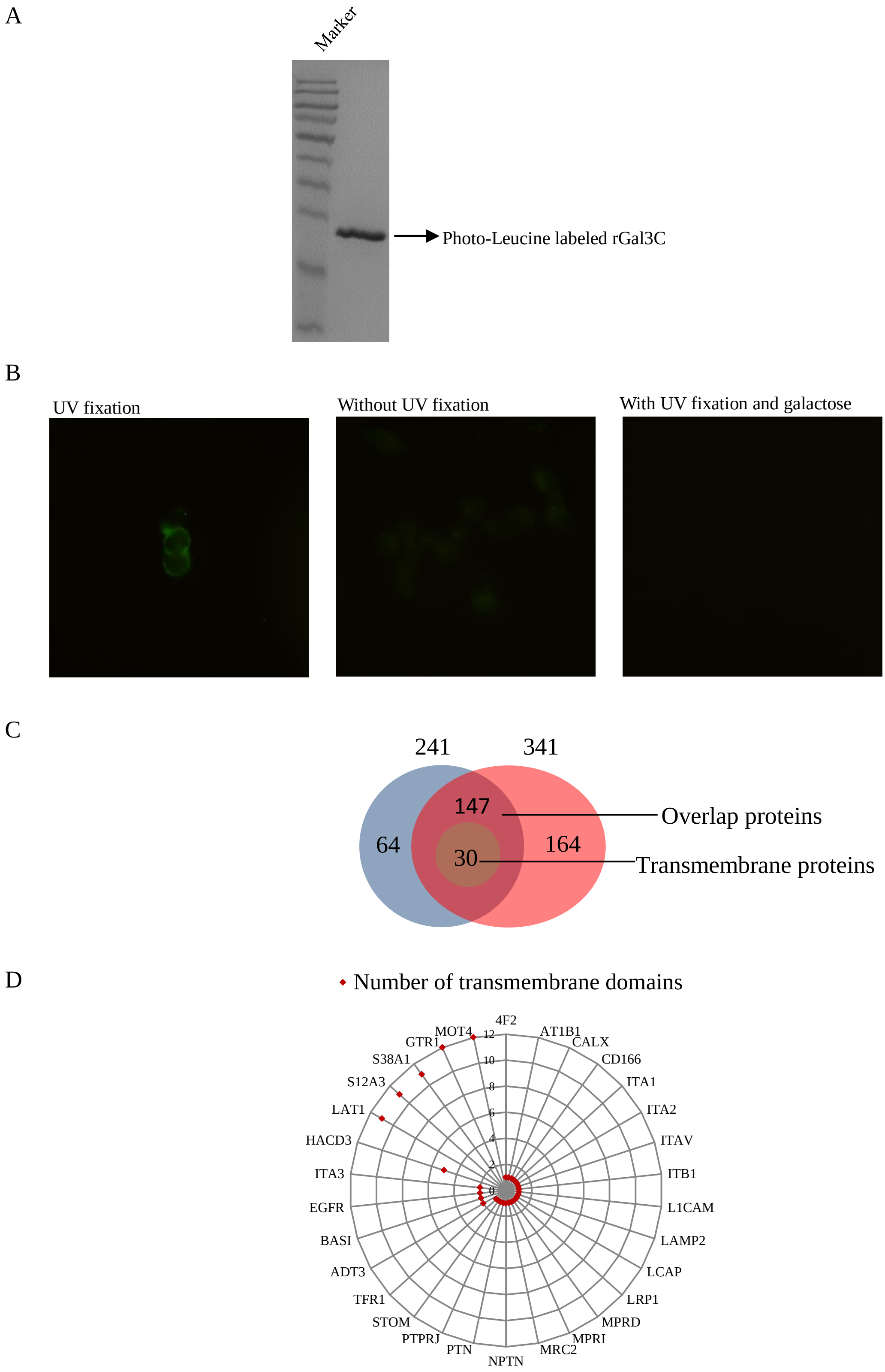

Supplementary Figure S3. (A) The Photo-Leucine labeled rGal3C was detected by 12% SDS-PAGE. (B) The binding of Photo-Leucine labeled rGal3C to HepG2 cell surface with UV fixation, without UV fixation or with UV fixation in the presence of lactose. (C) Overall 241 and 341 proteins were identified in two independent repeated experiments respectively, and 177 were repeated identified. In the 177 overlap proteins, 30 transmembrane proteins analyzed by TMHMM

Server v2.0 were considered as potential receptors of rGal3C. (D) A radar map displayed the number of transmembrane domians of the 30 potential receptors of rGal3C.

Supplementary table S1 List of up-regulated proteins

**Protein IDs**	**Gene names**	**unique peptide number**		**SILAC ratio**	
		H/L	L/H	H/L	L/H
**P09455**	RBP1	1	2	5.46	3.23
**Q9UJX2**	CDC23	3	1	1.31	3.05
**P31949**	S100A11	2	2	5.16	2.43
**Q9NX74**	DUS2	2	1	1.08	2.30
**O95470**	SGPL1	9	10	1.13	2.05
**Q6PUV4**	CPLX2	4	2	2.93	2.05
**Q14764**	MVP	8	5	2.54	2.03
**P37802**	TAGLN2	3	2	1.78	1.97
**O75746**	SLC25A12	3	2	1.03	1.87
**Q9BQL6**	FERMT1	2	3	1.72	1.86
**P09382**	LGALS1	4	4	2.87	1.85
**P15559**	NQO1	8	9	1.29	1.84
**Q56P03**	EAPP	1	2	1.01	1.81
**P01011**	SERPINA3	6	6	3.21	1.81
**P52943**	CRIP2	3	2	1.47	1.79
**O43301**	HSPA12A	2	2	1.78	1.77
**Q92887**	ABCC2	10	9	1.01	1.76
**Q9Y3A2**	UTP11L	3	2	1.01	1.76
**P38571**	LIPA	3	6	1.11	1.74
**P31947**	SFN	8	7	1.45	1.72
**P53634**	CTSC	7	6	1.12	1.68
**Q14914**	PTGR1	4	4	1.07	1.68
**O14908**	GIPC1	2	2	1.07	1.66
**Q8N1I0**	DOCK4	3	2	1.04	1.65
**P56747**	CLDN6	1	3	2.08	1.63
**Q9UGH3**	SLC23A2	1	1	1.23	1.63
**P10644**	PRKAR1A	9	8	1.13	1.61
**O60443**	DFNA5	6	5	1.10	1.59
**Q99519**	NEU1	4	3	1.91	1.54
**O75312**	ZPR1	5	3	1.06	1.54
**Q8IYU8**	MICU2	5	3	1.19	1.53
**Q00653**	NFKB2	3	3	1.19	1.53
**P06280**	GLA	2	3	1.03	1.53
**P36955**	SERPINF1	5	7	1.19	1.52
**P48506**	GCLC	10	12	1.14	1.51
**O60678**	PRMT3	2	3	1.21	1.51
**Q13509**	TUBB3	4	4	2.01	1.46
**P13284**	IFI30	2	2	1.78	1.44

**P00966**	ASS1	10	11	1.71	1.39
**P60981**	DSTN	7	9	1.50	1.38
**P01009**	SERPINA1	17	17	1.54	1.35
**O60218**	AKR1B10	16	16	2.31	1.33
**Q16658**	FSCN1	12	12	2.02	1.31
**P23368**	ME2	3	2	1.56	1.29
**P04792**	HSPB1	5	2	2.56	1.23
**Q16643**	DBN1	6	6	1.72	1.20
**Q13501**	SQSTM1	6	2	2.59	1.18
**P21333**	FLNA	66	67	1.54	1.16
**Q01995**	TAGLN	9	6	5.26	1.15
**Q9NR12**	PDLIM7	6	4	1.73	1.15
**Q99536**	VAT1	10	12	1.57	1.15
**Q9NRZ9**	HELLS	5	8	1.53	1.15
**Q14807**	KIF22	4	5	1.56	1.14
**Q8IYB7**	DIS3L2	3	4	1.57	1.14
**P01034**	CST3	3	2	1.55	1.13
**Q16881**	TXNRD1	12	14	1.79	1.13
**P67936**	TPM4	5	4	1.50	1.12
**Q9HC07**	TMEM165	2	2	1.68	1.11
**Q7Z406**	MYH14	3	2	1.83	1.10
**P51948**	MNAT1	1	1	1.67	1.10
**P52292**	KPNA2	6	6	1.85	1.10
**Q5TFE4**	NT5DC1	8	8	1.69	1.10
**P78504**	JAG1	3	2	1.68	1.09
**P46821**	MAP1B	3	3	1.93	1.09
**Q9UK76**	HN1	2	1	1.77	1.08
**P42575**	CASP2	2	1	1.53	1.07
**P35579**	MYH9	44	33	1.91	1.06
**P02794**	FTH1	3	2	1.91	1.05
**Q9H0A8**	COMMD4	6	2	1.52	1.05
**Q01664**	TFAP4	1	2	1.68	1.04
**Q9UPY5**	SLC7A11	1	2	2.47	1.04
**Q9ULT8**	HECTD1	11	3	1.63	1.03
**P04035**	HMGCR	6	2	2.50	1.03
**O14530**	TXNDC9	4	4	1.50	1.02
**Q9Y371**	SH3GLB1	6	2	1.70	1.01
**P31153**	MAT2A	5	2	1.60	1.01
**P06400**	RB1	5	3	1.58	1.01
**P46013**	MKI67	17	15	1.63	1.00
**Q99439**	CNN2	7	6	1.74	1.00

Supplementary Table S2 List of down-regulated proteins

Protein IDs	Gene names	unique peptide number		SILAC ratio	
		H/L	L/H	H/L	L/H
**Q08AH3**	ACSM2A	2	3	0.55	0.22
**Q92597**	NDRG1	9	9	0.37	0.38
**P10909**	CLU	11	6	0.29	0.53
**P48029**	SLC6A8	3	3	0.71	0.25
**P37059**	HSD17B2	5	6	0.86	0.21
**P08833**	IGFBP1	3	4	0.58	0.32
**P05121**	SERPINE1	9	12	0.59	0.34
**P0C7P0**	CISD3	1	2	0.61	0.33
**P23141**	CES1	26	27	0.95	0.27
**P08319**	ADH4	11	9	0.62	0.43
**P02671**	FGA	9	8	0.42	0.64
**Q06033**	ITIH3	6	2	0.32	0.85
**P35575**	G6PC	1	1	0.30	0.94
**P21549**	AGXT	9	9	0.84	0.35
**O00584**	RNASET2	7	9	0.46	0.64
**Q16877**	PFKFB4	5	4	0.57	0.54
**Q13740**	ALCAM	6	7	0.74	0.41
**Q02338**	BDH1	8	7	0.50	0.63
**P02787**	TF	30	28	0.61	0.53
**Q6UWM9**	UGT2A3	8	6	0.67	0.48
**Q4G0N4**	NADK2	6	5	0.60	0.54
**Q8N6C5**	IGSF1	14	8	0.46	0.72
**P55268**	LAMB2	6	4	0.70	0.49
**P13674**	P4HA1	15	17	0.52	0.66
**P07148**	FABP1	9	8	0.61	0.58
**Q9Y646**	CPQ	4	3	0.63	0.57
**P52569**	SLC7A2;SLC7A1	2	1	0.67	0.55
**P56181**	NDUFV3	1	1	0.43	0.88
**P10646**	TFPI	6	6	0.58	0.65
**Q9C0E8**	LNP	7	4	0.60	0.65
**Q8TEQ8**	PIGO	2	1	0.50	0.77
**P51687**	SUOX	2	1	0.74	0.54
**P09972**	ALDOC	9	10	0.64	0.62
**Q9BQE5**	APOL2	4	5	0.82	0.49
**Q93088**	BHMT	6	5	0.48	0.84
**Q9UDR5**	AASS	15	8	0.70	0.58
**Q2QL34**	MPV17L	2	2	0.73	0.56
**Q02252**	ALDH6A1	16	16	0.62	0.66
**Q969G6**	RFK	2	1	0.70	0.59
**Q9UN36**	NDRG2	7	5	0.63	0.66
**Q96I45**	TMEM141	2	1	0.64	0.66

**Q9UBM7**	DHCR7	3	2	0.47	0.90
**P16591**	FER	5	2	0.81	0.53
**Q96EY8**	MMAB	4	3	0.61	0.71
**Q96A26**	FAM162A	6	4	0.66	0.65
**Q15120**	PDK3	1	1	0.60	0.73
**Q8NBP7**	PCSK9	3	4	0.74	0.59
**Q96HE7**	ERO1L	12	13	0.58	0.76
**P08754**	GNAI3	2	3	0.46	0.97
**P00450**	CP	8	5	0.75	0.60
**P28332**	ADH6	9	10	0.65	0.69
**Q16134**	ETFDH	8	6	0.76	0.59
**Q9BY49**	PECR	11	12	0.55	0.83
**P00734**	F2	9	4	0.49	0.93
**P16662**	UGT2B7	4	5	0.95	0.48
**P78549**	NTHL1	1	1	0.52	0.88
**Q86WA6**	BPHL	8	6	0.54	0.86
**Q16850**	CYP51A1	15	12	0.84	0.55
**Q00577**	PURA	5	4	0.71	0.66
**Q99424**	ACOX2	6	5	0.67	0.71
**Q9BPW8**	NIPSNAP1	7	9	0.65	0.73
**O00468**	AGRN	16	11	0.63	0.75
**P39060**	COL18A1	6	6	0.74	0.65
**O95479**	H6PD	7	4	0.63	0.77
**Q16762**	TST	4	5	0.63	0.77
**Q15118**	PDK1	2	1	0.96	0.51
**P05204**	HMGN2;HMGN3	2	2	0.86	0.57
**Q6JQN1**	ACAD10	2	2	0.55	0.88
**O14638**	ENPP3	3	5	0.59	0.84
**Q04446**	GBE1	9	11	0.65	0.77
**Q8N4Z0**	RAB42	2	1	0.78	0.64
**O00560**	SDCBP	1	5	0.53	0.94
**P00387**	CYB5R3	8	7	0.62	0.81
**Q9UKR5**	C14orf1	2	3	0.62	0.81
**O00469**	PLOD2	16	18	0.60	0.85
**Q96QD8**	SLC38A2	6	5	0.50	1.00
**P02679**	FGG	13	14	0.63	0.80
**Q969Z3**	MARC2	3	4	0.80	0.63
**O95573**	ACSL3	17	18	0.89	0.57
**P46019**	PHKA2	6	7	0.66	0.77
**O75600**	GCAT	5	3	0.80	0.64
**P31327**	CPS1	21	10	0.61	0.84
**Q7Z3D6**	C14orf159	2	1	0.59	0.87
**Q13011**	ECH1	10	8	0.65	0.79
**Q9NR19**	ACSS2	13	11	0.81	0.65

**P05026**	ATP1B1	9	8	0.66	0.81
**Q9Y5U8**	MPC1	1	1	0.61	0.87
**Q5T2W1**	PDZK1;PDZK1P1	9	8	0.57	0.94
**P04080**	CSTB	6	5	0.89	0.60
**P02771**	AFP	17	22	0.67	0.80
**Q13825**	AUH	3	5	0.63	0.85
**Q9H0W9**	C11orf54	6	9	0.58	0.93
**P50453**	SERPINB9	11	7	0.92	0.58
**Q9NPJ3**	ACOT13	3	4	0.64	0.85
**Q15345**	LRRC41	2	4	0.66	0.83
**Q53GQ0**	HSD17B12	12	14	0.59	0.93
**P07305**	H1F0	7	6	0.63	0.89
**Q9H6V9**	C2orf43	3	5	0.65	0.87
**P61225**	RAP2B	2	1	0.65	0.87
**P35914**	HMGCL	6	9	0.62	0.92
**Q8N4T8**	CBR4	4	3	0.59	0.97
**P53365**	ARFIP2	2	2	0.92	0.62
**Q96BR5**	COA7	4	2	0.66	0.89
**Q8TE04**	PANK1	1	3	0.93	0.63
**Q008S8**	ECT2L	1	1	0.94	0.64
**O00264**	PGRMC1	4	3	0.66	0.94
**Q96AB3**	ISOC2	3	3	0.66	0.96
**Q9P0M6**	H2AFY2	13	12	0.63	1.00
**Q9H7C9**	AAMDC	3	5	0.67	0.97

Supplementary Table S3 List of potential receptors of rGal3C

Protein ID	Gene Name	Unique peptides_1	Unique peptides_2	iBAQ_1	iBAQ_2
**P08195**	4F2	13	15	51388000	102170000
**P26006**	ITA3	10	12	8734800	28183000
**P56199**	ITA1	5	12	2471600	8907500
**Q01650**	LAT1	4	4	27689000	31059000
**P17301**	ITA2	3	4	868750	3969000
**P05026**	AT1B1	2	3	1045300	28864000
**P35613**	BASI	2	3	9130800	20833000
**P27824**	CALX	2	3	979220	2728000
**Q13740**	CD166	2	5	816130	4586500
**P00533**	EGFR	2	6	1298000	5759300
**P06756**	ITAV	2	5	145470	2540000
**P32004**	L1CAM	2	6	423470	5865000
**P13473**	LAMP2	2	2	1830100	8407500
**Q9UIQ6**	LCAP	2	1	398200	322830
**P02786**	TFR1	2	1	352510	316730
**P12236**	ADT3	1	1	1446800	13671000
**P11166**	GTR1	1	2	2255600	3853300
**Q9P035**	HACD3	1	2	1331300	7679700
**P05556**	ITB1	1	5	0	11314000
**Q07954**	LRP1	1	1	53056	93367
**O15427**	MOT4	1	1	15661000	11441000
**P20645**	MPRD	1	1	1747600	2610500
**P11717**	MPRI	1	8	333210	1091100
**Q9UBG0**	MRC2	1	1	167160	888230
**Q9Y639**	NPTN	1	2	430810	2348500
**P21246**	PTN	1	1	1882300	25552000
**Q12913**	PTPRJ	1	5	427780	2018200
**P55017**	S12A3	1	1	301770	446250
**Q9H2H9**	S38A1	1	2	3849200	9622400
**P27105**	STOM	1	3	859220	6564700
